# Rhinocerebral Mucormycosis Following COVID-19 Infection in Iran

**DOI:** 10.30699/IJP.2023.545953.2845

**Published:** 2023-06-20

**Authors:** Parisa Khorasani Esmaili, Shahriar Dabiri, Touraj Reza Mirshekari, Fatemmeh Nabi Pour, Ayeh Shamsadini, Hadi Eslami, Mohammadad Ali Damghani, Ali Asghar Arabi, Maryam Aamizadeh, Fatemeh Fani Maleki, Navid Gharaie, Shiva Pouradeli

**Affiliations:** 1 *Department of Pathology, Pathology and Stem Cell Research, Afzali Pour Medical Faculty, Kerman University of Medical Science, Kerman, Iran*; 2 *Department of Pathology, Shafa Hospital, Kerman University of Medical Science, Kerman, Iran *; 3 *Department of Otorhinolaryngology and Head & Neck Surgery, Shafa Hospital, Kerman University of Medical Science, Kerman, Iran*; 4 *Clinical Research Development Unit, Shafa Hospital, Kerman University of Medical Sciences, Kerman, Iran*

**Keywords:** Black fungal, COVID-19, Mucormycosis, Rhinocerebral, Iran

## Abstract

**Background & Objective:**

Mucormycosis (also called black fungus) is an opportunistic serious fungal infection caused by mucormycetes. It can occur in diabetes mellitus patients and other immunosuppressive conditions with recent predisposing factors such as maxillofacial surgery and corticosteroid usage.

**Methods:**

In this study, 14 patients were referred to the otorhinolaryngology or ophthalmology ward of Shafa Hospital (Kerman, Iran) with primary symptoms of nasal fullness and facial nerve dysfunction; they were admitted to the hospital to rule out the fungal infection. An endoscopic biopsy was taken from facial sinuses or orbit, and a microscopic evaluation was performed using hematoxylin and eosin (H&E) and periodic acid–Schiff (PAS) staining methods to rule out mucormycosis.

**Results:**

In the histopathological examination, broad-based nonseptate branching fungal hyphae were found in nasal sinuses through the endoscopic biopsy. Most of the patients had diabetes mellitus with a primary symptom of facial nerve palsy; also, most of them received corticosteroids (intravenous [IV] or intramuscular [IM] injection). All patients have recently been infected with COVID-19 (less than 1 month ago).

**Conclusion:**

COVID-19 infection might be a predisposing factor for many opportunistic infections, such as fungal elements); thus, the physician should be aware of the dosage and duration of corticosteroid therapy to prevent the development of these infections.

## Introduction

Mucormycosis is a fungal infection caused by mucormycetes. It can be fatal when it does not treat immediately. In India, the incidence of mucormycosis increased during the second wave of COVID-19 compared to the first wave. The predisposing factors of mucormycosis in COVID-19 patients are uncontrolled diabetes, the excessive prescription of corticosteroids for immunosuppression, and longtime hospitalization in the intensive care unit (ICU) (1).

Low oxygen (hypoxia), high glucose (diabetes, new-onset hyperglycemia, steroid-induced hyperglycemia), metabolic acidosis, diabetic ketoacidosis (DKA), high iron levels (increased ferritin), decreased phagocytic activity of white blood cells due to immunosuppression (SARS-CoV-2–mediated, steroid-mediated, or background comorbidities), and prolonged hospitalization with or without mechanical ventilators are among the factors that could increase the risk of mucormycosis infection in COVID-19 patients (1,2). Several studies have claimed that systemic corticosteroid therapy improves clinical outcomes and reduces mortality in hospitalized COVID-19 patients (3,4). Systemic inflammatory response and cytokine storm in COVID-19 lead to lung injury and systematic dysfunction. In contrast, in hospitalized COVID-19 patients who do not require supplemental oxygen, used systemic corticosteroids have not shown any benefits and may be susceptible to fungal infections (5,6).

Many studies have recommended a daily dose of 6 mg of dexamethasone. Two trials have suggested that a 12-mg corticosteroid dose be used in patients who require respiratory support. However, some clinicians may choose to administer a higher dose of dexamethasone to these patients. We should remind that currently, there is no data evaluating the safety and efficacy of using lower or higher doses of corticosteroids in combination with other immunomodulators to treat COVID-19 (7,8,9,10).

## Material and Methods

In this study, 14 patients who were hospitalized in the otorhinolaryngology section of Shafa Hospital (Kerman, Iran) from August to September 2021 andpresented with rhinocerebral mucormycosis and recent COVID-19 infection, were included in this study. Inclusion criteria were a positive endoscopic biopsy for mucormycosis and recent COVID-19 infection (confirmed by polymerase chain reaction [PCR] test). All patients had received at least a daily dose of 6 mg of dexamethasone according to the National Institutes of Health (NIH) protocol.

**Fig. 1:**
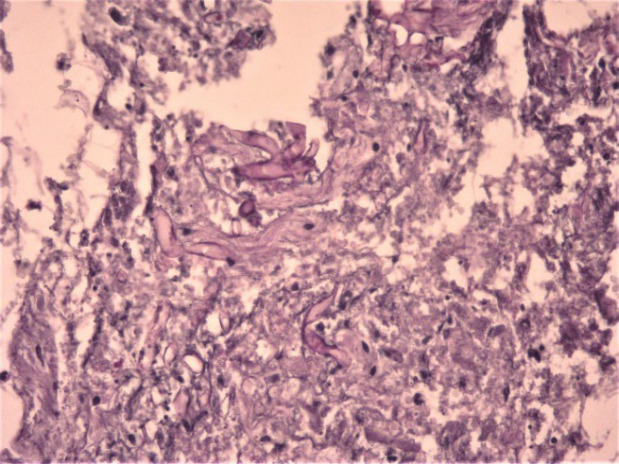
Fungal elements with right angle hyphae (H&E staining x100)

**Fig. 2:**
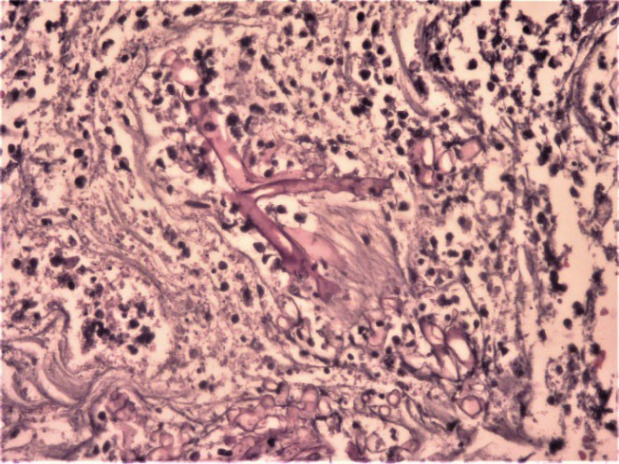
Fungal elements with right angle hyphae (H&E staining x100)

**Fig. 3:**
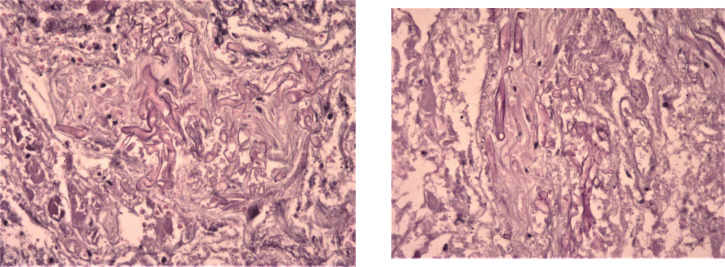
Fungal elements with P.A.S staining (x100)

In all the patients, clinical presentations suggested a pathological state in the paranasal sinuses. An endoscopic evaluation was done to rule out the fungal infection. A biopsy and, if needed, paranasal sinus debridement (especially for necrotic sites) were done immediately. In some cases, if clinically indicated, an orbital biopsy was taken as well. Biopsies received in neutral buffered formalin solution (10%), serial sections from biopsies which stained by hematoxylin and eosin (H&E) for confirmation periodic acid–Schiff (PAS) was done. All the histopathological slide cases were reviewed separately by 2 different pathologists (Figures 1, 2, and 3)). 

When the biopsy confirmed mucormycosis infection, debridement was done again, and intravenous (IV) antifungal drugs (liposomal amphotericin B) were given.

## Results

Fourteen patients with a mean age of 56 ± 13.6 years (28-70) participated in this study. Among them, 11 (78.6%) were women and 3 (21.4%) were men. Eleven patients had diabetes mellitus (78.6%) as comorbidity and a risk factor for mucormycosis, of whom 2 had no significant past medical history, and 1 was a known case of acute myeloid leukemia (AML; immunocompromised state). Increased hypertension (HTN; 28.6%) and hyperlipidemia (21.4%) were observed in patients.

The most clinical presentation was periorbital edema and ophthalmoplegia. The recent chest computed tomography (CT) scan that was done for COVID-19 showed no significant percentage of involvement in most patients; in 2 patients, more than 40% involvement was noticed. Most patients mentioned recent IV or intramuscular (IM) corticosteroid usage (dexamethasone or hydrocortisone, 100 mg); the mean dose of corticosteroids was 5.5 mg/dose. One of them was in the intensive care unit (ICU) a month ago. The white blood cell (WBC) count on the first day of hospitalization was from 4.6 to 40.6 × 10^9^/UL with a mean of 13.678 × 10^9^/UL. Two patients had recent DKA and metabolic acidosis.

The patients’ characteristics included chest CT scan findings (for COVID-19 infection), underlying disease, recent IV corticosteroids, mucormycosis risk factor, ICU hospitalization, WBC count on the first day of hospitalization, and history of recent DKA. 

Also, collected data included hemoglobin A1c (HbA1c; to evaluate blood sugar control), initial clinical presentation of fungal infection, addiction, site of *Mucor* involvement (paranasal sinus, palate, orbit, or cerebral areas), day of *Mucor* diagnosis after COVID-19, orbital enucleation, dose of liposomal amphotericin B, and vaccination dosage (against COVID-19). Most patients (78.6%) did not have a complete vaccination status.

Most of the patients with high HbA1c, showing poor control of diabetes mellitus with a mean average of 8.63 (a range of 5.4-12), and 93% of patients did not mention a history of severe dyspnea requiring intubation. Only a few of them used O_2_ masks or cannulas. One patient was a smoker, 3 (21.4%) were opium addicts, and 10 (71.4%) had no history of drug addiction (Table 1).

The most common sites of fungal involvement were the nasal sinuses. Two patients’ primary presentations of fungal infection started at the same time as the COVID-19 presentation. In our cases, *Mucor* was present from the first day to 30 days after the COVID-19 infection.

The mean amount of liposomal amphotericin B that patients received was 39.4 vials (1970 mg), at least 3 vials (a total dosage of 150 mg), and a maximal dosage of 83 vials (a total dosage of 4150 mg during hospitalization). Each vial dosage was 50 mg.

Orbital enucleation was done in 5 patients. Only 3 (21.4%) patients expired, and the mortality rate was approximately 21%, even with IV antifungal injection and multiple sinus debridement. For all the cases, debridement was done multiple times (at least 2 times), and a biopsy was taken each time.

Histopathological examination was performed by H&E and PAS staining methods using a light microscope, showing necrotic tissue, hemorrhage, acute and chronic inflammatory cell infiltrate with leukocytoclastic vasculitis, and infiltration of broad-based fungal elements with nonseptate branching hyphae (right angle) that invade vascular tissue or infiltrated into the necrotic site. The common sites of involvement were paranasal sinuses. Orbital enucleation was performed for patients whose orbital biopsy was positive for mucormycosis or who had cerebral symptoms that definitely showed orbital involvement. Mucormycosis specimens were reported in all 5 orbitals. Only 1 patient showed another fungal element, which was similar to *Aspergillus* with septate branching hyphae (Tables 2, and 3).

After data analysis in logistic regression, any of variables such as diabetes mellitus, recent DKA, corticosteroid dose/day, immunocompromised state cannot predicted outcomes included expire or alive, cure and orbital enucleation (Tables 4 and 5).

**Table 1:** The mean and SD of quantitative variables

	Minimum	Maximum	Mean	SD
Age	28	70	56.00	13.621
First day WBC	4700	40600	13678.57	10414.131
HbA1c	5.4	12.0	8.636	2.4111
Amphotericin/dose	3	84	39.43	30.409
Day of mucor after COVID	0	30	12.79	9.250
Corticosteroid /dose (mg)	8	348	79.54	88.303
Corticosteroid Day	1	14	6.46	3.908
Total dose (mg)	8	3132	661.54	871.570
Mean daily use	.0	38.6	11.214	10.3220

**Table 2:** The frequency of the two category variables in the patients

Variable	Category	Frequency	Percent
DKA	Yes	4	28.6
	No	10	71.4
ICU	Yes	1	7.1
	No	13	92.9
Brain Involvement	Yes	5	35.7
	No	9	64.3
Orbit enucleation	Yes	5	35.7
	No	9	64.3
Debridement	Yes	100	100.0
	No	0	0
addiction	Yes	4	28.6
	No	10	71.4
Vaccine	Yes	3	21.4
	No	11	78.6
Alive	Yes	11	78.6
	No	3	21.4

**Table 3:** The chest CT, corticosteroid therapy dosageand clinical presentation of the patients

No	Chest CT	Corticosteroid Day/dose	Total corticosteroid dose	Mean daily corticosteroid use	Clinical presentation
1	Not involved	4 dose dexamethasone	32 mg in 4 days	8 mg	Ophtalmoplegia periorbital pain
2	Not involved	10 dose dexamethasone	80 mg in 10 days	8 mg	Periorbital edema
3	Less than 10%	3 dose dexamethasone	24 mg in 3 days	8 mg	Periorbital edema ophtalmoplagia, facial paresthesia
4	15%	11 dose dexamethasone	88 mg in 11 days	8 mg	Facial paralysis, ophtalmoplagia
5	No	6 dose dexamethasone 3 dose hydrocortisone 100MG	348 mg in 9 days	38.6 mg	Bilateral periorbital edema
6	60-70%	14 dose dexamethasone	112 mg in 14 days	8 mg	Ophtalmoplagia , blindness, facial paralysis
7	30% involved	1 dose dexamethasone	8 mg in 1 day	8 mg	Headache
8	Massive pleural effusion	5 dose dexamethasone	40 mg in 5 days	8 mg	Ophtalmoplagia periorbital pain, blindness
9	20%	6 dose dexamethasone	48 mg in 6 days	8 mg	Orbital pain, ophtalmoplagia, facial paralysis
10	Lung fissure involvement	3 dose dexamethasone 1 dose hydrocortisone	124 mg in 4 days	31 mg	Proptosis periorbital edema blindness Frozen eye
11	40-50%	-	0	0	Ophtalmoplagia, headache
12	less than 10%	3 dose dexamethasone prednisolone 5 daily	74 mg in 10 days	7.4 mg	Orbital pain ,blindness ,headache
13	Not involved	3 dose dexamethasone	24 mg in 3 days	8 mg	Frozen eye blindness Broca aphasia
14	Less than 5%	4 dose dexamethasone	32 mg in 4 days	8 mg	Fever, Epistaxis, Perinasal pain and fullness

**Table 4.** Frequency of the multi-category variables in the patients

Mucor mycosis Risk factor			DM					78.6		
			Immunocompromised					7.1		
			-					14.3		
O2			canula					21.4		
			Intubation					7.1		
			mask					28.6		
			No					42.9		
Site of involvement			**sinus**					100.0		
			**Orbit**					35.7		
			**Palate**					7.1		
			**cerebral**					28.6		
			**Buccal**					7.1		
			**mucosa**					7.1		
Underlying Disease			**DM**					78.6		
			**HTN**					28.6		
			**HLP**					21.4		
			Other disease (SINGLE KIDNEY, ASTHEMA, EF:30%, AML)					26.7		
Clinical presentation			Periorbital edema					42.9		
			blindness					35.7		
			Ophtalmoplegia					50.0		
			Orbital					14.3		
			pain					35.7		
			edema					28.6		
			headache					21.4		
			facial paralysis					21.4		
			Frozen eye					14.3		
			Others (facial paresthesia, Bilateral, Proptosis, Broca aphasia , Fever, Epistaxis, Perinasal, fullness)					57.14		
	Risk factors					
No	DM	Corticosteroid dose/day		Recent COVID	Immunocompromised	Recent DKA	Cure*		Orbital enucleation	expire
1	Yes	8mg		Yes		Yes	Yes			
2	No	8mg		Yes			Yes			
3	Yes	8mg		Yes					Yes	
4	Yes	8mg		Yes			yes			
5	Yes	38.6mg		Yes			Yes			
6	Yes	8mg		Yes		Yes			Yes	
7	Yes	8mg		Yes		Yes	Yes			
8	No	8mg		Yes					Yes	Yes
9	Yes	8mg		Yes						Yes
10	Yes	31mg		Yes		Yes			Yes	yes
11	Yes	0		Yes					Yes	
12	Yes	7.4mg		Yes			Yes			
13	Yes	8mg		Yes			Yes			
14	No	8mg		Yes	Yes		Yes			

*Cure include complete cure or facial nerve paralysis group

**Table 5:** The risk factors and out comes

		P-value	Exp(B)
Step 1^a^	DM	0.406	0.230
	Corticosteroid dose/day	0.336	1.062
	Recent DKA	0.702	1.888
	Constant	0.374	0.309
Expire or alive			
Step 1^a^		**P-value**	**Exp(B)**
	DM	0.752	0.631
	Corticosteroid dose/day	0.745	1.019
	Recent DKA	0.790	0.711
	Constant	0.678	1.722
Cure		**P-value**	**Exp(B)**
	DM	0.896	0.823
	Corticosteroid dose/day	0.870	0.990
	Recent DKA	0.474	2.573
	Constant	0.641	0.541

Orbital enucleation

## Discussion

Previous studies conducted on mucormycosis infection and predisposing factors have only assessed diabetes mellitus, serum iron level, maxillofacial surgery, recent corticosteroid therapy, and immunocompromised state; however, during the COVID-19 pandemic, mucormycosis cases increased. Accordingly, we tried to find the relationship between COVID-19 infection and mucormycosis as a hypothesis.

This study can show clues for the association between COVID-19 and mucormycosis infection in the 5th wave of COVID-19 infection in Kerman/Iran. In most COVID-19 patients, WBC levels and lymphocytes decreased; in addition, immunosuppressive corticosteroid therapy could reduce the phagocytic activity of neutrophils and macrophages (our study showed 4.6 to 40.6 × 10^9^/UL WBC with a mean of 13.678 × 10^9^/UL).

On the other hand, corticosteroid therapy causes increased blood sugar (especially in diabetic patients), worsening their immune responses.

Increased cytokine levels (such as interleukin 6 and ferritin levels of COVID-19 patients). Recent studies have claimed that COVID-19 caused high blood sugar levels, low O_2_ levels, and suppressed immune system, all of which are risk factors that make a good environment for opportunistic infections like mucormycosis.

Although the US Centers for Disease Control and Prevention suggested IV antifungal drugs (such as amphotericin B) and surgical interventions for mucormycosis infection, the mortality rate (54%) is still high (11,12). 

Malpractice and/or self-treatment by steroids, antibiotics, and zinc drugs, which are increased during the COVID-19 pandemic) may have promoted the intestinal dysbiosis of normal flora and induced immune suppression (12,13,26).

In COVID-19, the first factor that increases the risk of invasive fungal infection could be alveolo-interstitial damage. In a study, Bahadori *et al.* reported that histopathological evaluation on different tissue from COVID patients showed variable degrees of vasculitis (degenerative, endothelial cell necrosis, and inflammatory cells in the vessel wall with fibrinoid necrosis). Tissue damage can cause interstitial acute inflammatory cell reaction with degenerative reaction and necrosis. Endothelial cell degeneration at the vessel wall was confirmed by CD34 and factor VIII immunohistochemistry staining (14).

Risk factors for mucormycosis infection include diabetes mellitus, previous respiratory pathology, immunosuppressive states like steroid therapy or usage of monoclonal antibodies, and broad-spectrum antibiotics.

Current COVID-19 guidelines in India recommend 0.5-1 mg/kg/day of IV methylprednisolone (3 days) in moderate cases and 1-2 mg/kg/day in severe cases; however, the NIH recommends dexamethasone (6 mg/day) for a maximum of 10 days in ventilated patients––but not in mild cases (15,16). In this study, at least 8 mg of corticosteroid was administered for COVID-19 patients (mean: 79.5 mg/day).

The most common presentation was ophthalmoplegia, followed by orbital cellulitis and pan sinusitis in imaging. IV liposomal amphotericin B was administered to all patients for an average of 18.93 days. The HbA1c ≥8 is a predictor factor for cerebral involvement (17).

The median interval between COVID-19 disease and diagnosis of mucormycosis was reported as 7 days (range: 1-37 days) (18); all patients in this study presented mucormycosis with a mean of 12.7 days after COVID-19 infection.

Singh *et al.* (2021) conducted a study on 101 COVID-19 patients with mucormycosis. They claimed that most mucormycosis patients were male (78.9%) with either active or recovered COVID-19 infection. Diabetes mellitus was mentioned in 80% of cases as a predisposing factor, and 14.9% had DKA. In the current study, most cases had diabetes mellitus. Approximately 76.3% of COVID-19 patients received corticosteroids.

Nasal and paranasal sinuses were the most common sites of involvement (19,27,28). The primary site of *Mucor *infection was the sinuses in our study, followed by other sites such as the palate, orbit, and cerebral.

In a systematic review by Vaezi *et al.* in Iran, all mucormycosis cases were reported from 1990 to 2015 (25 years). Diabetes was the most common underlying disease, and 22.4% of the patients mentioned solid organ or bone marrow transplantation. The mortality rate in mucormycosis cases was 40.8%, with the highest mortality rate in patients diagnosed with disseminated infection (75%) (20).

Orbital apex syndrome is a rare presentation of mucormycosis that include ophthalmoplegia and rapid vision loss, involving cranial nerves II, III, IV, V, and VI and requiring anti-fungal and surgical debridement (21,24,25).

The humidity in the nasal cavity provides a suitable environment for the primary growth of fungi and then infection extends to the paranasal sinuses. The middle turbinate, middle meatus, and septum are the most common sites of mucormycosis infection. The infection almost extends to all surrounding tissues and then invades the brain via ethmoid sinuses, orbital apex, and bone erosion or angioinvasion.

Fungal hyphae invade blood vessels, causing endothelium damage, resulting in blood clot formation, ischemia, and necrosis. In rhinocerebral mucormycosis, invasion into the brain and orbit is through the involvement of sphenopalatine and internal maxillary arteries. The involvement of the internal carotid artery and cavernous sinus thrombosis is common in prolonged disease (22).

In Iran, Nashibi conducted a study on the epidemiology and treatment outcome of mucormycosis. They showed that the most prevalent season of mucormycosis was winter (40%); all the patients received amphotericin B, and 50% of the patients experienced surgical debridement (23). Our study was in the summer of 2021; all the patients presented the disease in the summer time, were residents in a dry city in Iran (Kerman) after COVID-19 infection, underwent debridement, and received an antifungal medication.

## Conclusion

All clinicians should be aware of opportunistic fungal infections in patients with COVID-19 (especially mucormycosis and its relationship with therapeutic corticosteroids); thus, corticosteroids should be administered at a low dose with a short-term use to prevent any opportunistic infections.

In this study whilethe iron level was not measured, it might contribute in the development of mucormycosis infection.

## Conflict of Interest

The authors declared no conflicts of interest.
